# The Right Tools for the Job: Evaluating Frameworks for Chemical Alternatives Assessment

**DOI:** 10.1289/ehp.124-A58

**Published:** 2016-03-01

**Authors:** Carrie Arnold

**Affiliations:** Carrie Arnold is a freelance science writer living in Virginia. Her work has appeared in *Scientific American*, *Discover*, *New Scientist*, *Smithsonian*, and more.

With the rise in green chemistry and growing concern over worker and consumer protection, businesses and regulatory agencies are increasingly looking to identify alternative chemicals for use in products and manufacturing processes. Alternatives assessment involves comparing the advantages and disadvantages of potential substitutes for toxic chemicals,^1^ and numerous agencies, nonprofits, and businesses have developed frameworks to help them conduct these analyses. In this issue of *EHP*, investigators review nearly two dozen of alternatives assessment frameworks to identify what’s working and what needs improvement in this rapidly advancing field.^2^

As governments around the world begin to require alternatives assessments for chemicals of high concern,^3^ the need for more robust decision-making capabilities is becoming apparent, says first author Molly Jacobs, a project manager at the Lowell Center for Sustainable Production. To determine the factors common to a high-quality alternatives assessment framework, as well as identify areas that require more work, Jacobs and colleagues identified 20 frameworks that have been published since 1990 and evaluated six core areas: hazard assessment, exposure characterization, life cycle impacts, technical feasibility assessment, economic feasibility assessment, and decision-making processes for reaching conclusions about alternatives.

**Figure:**
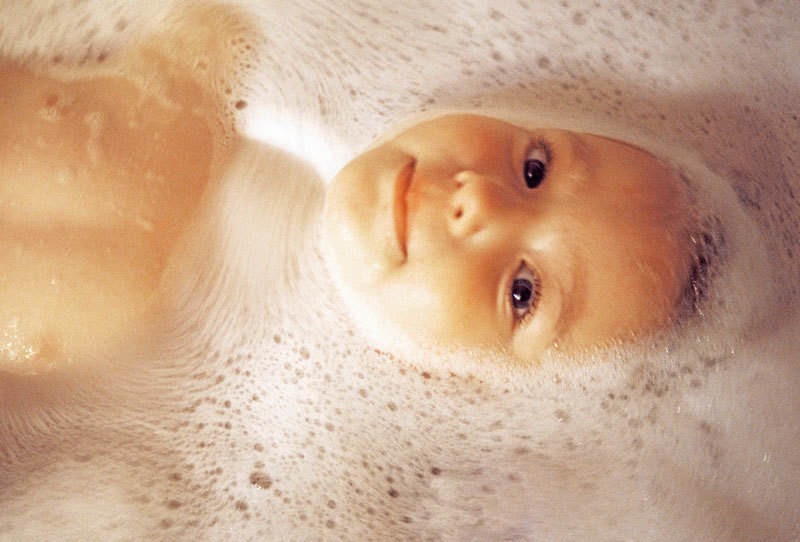
Identifying less-toxic alternatives to chemicals of concern ideally will lead to safer consumer products. © MacGregor & Gordon/Getty

The group’s analysis showed that all the assessment frameworks incorporated hazards, costs, and performance. However, they lacked consistency in ranking and scoring the hazards created by different chemicals, as well as in the end points they addressed and the methods and criteria used to reach conclusions.^2^

Some of the difficulty in comparing these frameworks is that they are designed to accommodate the specific needs of the parties using them. For instance, a small business will use quite a different framework than a large corporation or governmental agency, because the latter have the resources to do a much more in-depth assessment. “There is need for flexibility in the approach to using these alternatives assessment frameworks given differences in context—regulatory versus nonregulatory, government versus business, differences in business positions along the supply chain, etc.,” Jacobs explains.

Selecting an alternative isn’t always straightforward. Businesses need to factor in the cost of replacements as well as how a new chemical will fit into existing manufacturing processes, says Paul DeLeo, associate vice president for environmental safety at the American Cleaning Institute, which represents manufacturers of consumer cleaning products. “We’re doing alternatives assessment every day—it’s part of product design,” DeLeo says. But it’s a complicated process with a lot of moving parts. “We need to distill the process down into more manageable segments,” he says.

Suitable alternatives don’t have to be risk-free, just lower-risk, and the risk needs to be manageable with appropriate protections. “There are always trade-offs to make, such as determining whether it’s better to use a little of a chemical that’s more dangerous or a lot of one that’s more moderate,” says Igor Linkov, a risk and decision scientist for the U.S. Army Engineer Research and Development Center. Linkov was not involved with the review.

At the same time, says coauthor Joel Tickner, an environmental health scientist at the University of Massachusetts Lowell, “we need to make sure that we don’t jump out of the frying pan and into the fire.” As one example of this, the authors point to 1-bromopropane (1-BP, also called *n*-propyl bromide). In the late 1990s 1-BP was marketed as an alternative for banned solvents such as 1,1,1-trichloroethane. Some companies also began to substitute 1-BP for other commonly used solvents. But in the years that followed, a number of published case reports described neurotoxic effects in workers exposed to 1-BP,^4^^,^^5^^,^^6^ and 1-BP is now designated as reasonably anticipated to be a human carcinogen.^7^ 1-BP became the epitome of what the Occupational Safety and Health Administration calls “regrettable substitutions.”^8^

Given the emphasis on green chemistry, the field of alternatives assessment likely will only continue to pick up momentum, Linkov says. But the results of the new analysis indicate that ongoing interdisciplinary research will be required to take alternatives assessments to their highest potential.
